# Becoming a *normal* guy: Men making sense of long-term bodily changes following bariatric surgery

**DOI:** 10.3402/qhw.v10.29923

**Published:** 2015-12-04

**Authors:** Karen Synne Groven, Paul Galdas, Kari Nyheim Solbrække

**Affiliations:** 1 Faculty of Medicine, Institute of Health and Society, University of Oslo, Oslo, Norway; 2 Institute of Physiotherapy, Oslo and Akershus University College of Applied Sciences, Oslo, Norway; 3 Reader in Nursing, Department of Health Sciences, University of York, York, United Kingdom

**Keywords:** Gastric bypass surgery, men, interviews, body, masculinity

## Abstract

**Background:**

To date, research on bodily changes following bariatric surgery has focused predominantly on women, leaving the long-term experience of men relatively unexplored. In this paper, we draw on interviews with men who have undergone an irreversible gastric bypass procedure to explore their bodily changes more than 4 years post-surgery. We apply a phenomenological framework that draws on Leder's perspectives on the “disappearing” and “dys-appearing” body, combined with a gender-sensitive lens that draws on Connell's theory of hegemonic masculinity and Robertson's conceptions of embodied masculinity.

**Findings:**

Our principal finding was that the men negotiated their bodily changes following bariatric surgery in profoundly ambivalent ways. Although they enthusiastically praised the surgery for improving their health, self-esteem, and social functioning, they also emphasized their efforts to cope with post-surgical side effects and life-threatening complications. Our analysis elaborates on their efforts to adjust to and come to terms with these changes, focusing on *episodes of hypoglycemia*, *severe pain and internal herniation*, and *the significance of physical activity and exercise*.

**Conclusions:**

Our findings point to the need to acknowledge men's ways of making sense of profound and ongoing bodily changes following bariatric surgery and how these negotiations are closely intertwined with masculine ideals of embodiment and social value.

Bariatric surgery is increasingly offered to individuals struggling with obesity and, while numerous research studies have documented its effect, there is a paucity of knowledge about men's experience in the years following bariatric surgery. Utilizing Leder's phenomenology of the body and Connell's perspective of hegemonic masculinity as theoretical frameworks, in this article we present an in-depth examination of the lived experience of five Norwegian men who have undergone gastric bypass surgery. Worldwide, gastric bypass is the most commonly performed bariatric procedure (Colquitt, Pickett, Loveman, & Frampton, 2014; Kulick, Hark, & Deen, 2010). This pattern is reflected in Norway, where nearly 90% of all bariatric surgeries are gastric bypasses (Kristinsson, 2008). The procedure reduces the stomach capacity radically and, unlike restrictive surgeries, it also reroutes part of the small intestine, causing malabsorption. The combination of restriction and malabsorption usually results in greater weight loss compared to restrictive procedures, such as the gastric banding procedure. Consequently, gastric bypass is considered by many surgeons to be the “gold standard” of bariatric surgery (Colquitt et al., 2014; Kulick et al., 2010); although others contest this, arguing that duodenal switch is more effective in terms of treating type 2 diabetes (Natvik, 2015).

Research interest in bariatric surgery has increased in tandem with the growing obesity epidemic worldwide. According to the World Health Organization, obesity rates have more than doubled since the 1980s, and recent estimates suggest that more than 1.9 million adults 18 years and older can be classified as overweight or obese. Recent estimates also suggest that 42 million children under the age of 5 can be classified as overweight or obese. This growing epidemic is widely recognized as one of the greatest threats to public health. Indeed, most of the world's population lives in countries where overweight and obesity kill more people than underweight.^1^ Obesity is associated with a variety of serious health problems, including heart/vascular diseases, type 2 diabetes, fatty liver disease, kidney disease, and certain kinds of cancer. In addition, obesity is associated with musculoskeletal pain, obstructive sleep apnea, emotional distress, and depression affecting individuals’ (health-related) quality of life in profound ways (Danielsen, 2015; National Institutes of Health, 2012; Malterud & Tonstad, 2009; Speck, Bond, Sarwer, & Farrar, 2014; Natvik, 2015).

Bariatric surgery is currently regarded as the most effective medical intervention for obesity. Compared to conservative lifestyle interventions, which are associated with disappointing long-term results, bariatric surgery is associated with significant weight loss as well as positive impacts on obesity-related diseases and health-related quality of life (Adams et al., 2012; Buchwald & Oien, 2013; Colquitt et al., 2014; Hofsø, 2011; Natvik, 2015; Puzziferri et al., 2014). Positive results have also been documented in terms of mental health and psychosocial functioning, especially during the first two post-surgical years (Adams et al., 2012; Assimakopoulos et al., 2011; Nasjonalt Kunnskapssenter for Helsetjenesten, 2014).

Like other major surgical interventions, bariatric surgery is associated with risk. Although the severity of complications has decreased as a result of improvements in technology, 3–5% of bariatric patients have been reported to need reoperations due to acute complications such as internal herniation, gastric anastomosis leak, and pulmonary and venous embolism (Groven, 2014; Hadam, Somers, & Chand, 2011; Hofsø 2011; Mala & Kristinsson, 2013; Nasjonalt kunnskapssenter for helsetjenesten, 2014; Natvik, 2015). In addition, bariatric surgery is also associated with side effects such as excess skin, malnutrition, and various intestinal problems (Aasheim et al., 2007; Hadam et al., 2011; Light, Arvanitis, Abramson, & Glasberg, 2010). Many types of bariatric surgery require long-term supplementation with vitamins and iron, and patients often have a very restricted liquid diet in the immediate weeks after surgery (Colquitt et al., 2014). The most common side effect following gastric bypass surgery, affecting approximately 80% of patients, is “dumping.” Symptoms associated with dumping include irregular heartbeat, diarrhea, dizziness, and nausea (Groven, Engelsrud, & Råheim, 2012; Tack et al., 2009). Recent studies indicate that 20–30% of patients undergoing gastric bypass also report *late dumping* (also termed *hypoglycemia*). Whereas early dumping usually occurs within 10–30 min after eating, late dumping is reported to occur 2–3 h after eating (Kulick et al., 2010).

Although the physical sequelae of bariatric surgery have been widely studied, individuals’ experience of living with the side effects and complications following bariatric surgery have rarely been the focus of attention. Little knowledge also exists regarding the long-term health effects of bariatric surgery. These gaps in the current evidence base have prompted Norwegian health authorities to call for more research on the long-term implications of bariatric surgery, including more qualitative studies on men's and women's experiences following such procedures. Taking a lead from health authorities in Denmark, the Norwegian Council for Priority Settings in Health Care have also recommended restricted access to bariatric surgery (Groven, & Hofmann, 2015; Natvik, 2015):

Bariatric surgery results in large and rapid weight loss, but can also result in complications and side effects. The patients often need lifelong follow up from the health service. Long-term effects are not well known, and we do not know whether bariatric surgery improves survival for the patients. We also know little about how morbidity and quality of life are affected in the long term …. The council underlines that both private and public clinics must offer their patients good information and guidance about the risks of bariatric surgery and uncertainty about long-term effects.^2^

In our exploration of men's lived experience following bariatric surgery our focus is on a “minority group” in this field of research. Indeed, resent estimates indicate that 70–80% of participants in bariatric studies are women—a pattern that is also reflected in the way bariatric surgery is debated and portrayed in popular culture (Jakobsen, Hofsø, Røislien, Sandbu, & Hjelmesæth, 2010; Martin, Beekly, Kjorstad, & Sebesta, 2010; Newhook, Gregory, & Twells, 2015). Feminist scholars have critically examined the gendering of bariatric surgery in media and marketing representations of the procedure, pointing out significant contrasts in men's and women's accounts. Whereas women's accounts tended to emphasize changes in body image, marital status, and self-esteem, men's accounts highlighted their improved health and physical strength, typically featuring men in athletic wear as they ran a marathon or played soccer (Groven & Braithwaite, 2015; Glenn, Champion, & Spence, 2012; Glenn, McGannon, & Spence, 2012; Salant & Santry, 2006).

Recent studies have further highlighted how health behavior based on different gender roles may play a role in obesity and weight management, especially in relation to health effects and weight loss. In a review of the literature, Robertson et al. (2014) found that, while men were less likely than women to seek help for obesity problems, once enrolled in weight loss programs they were less likely to drop out. However, it should be noted that studies such as that of Robertson and colleagues apply a biomedical approach, drawing on statistics to explore the measurable effects of dietary and exercise interventions. There remains a gap in the literature regarding the ways in which men experience and relate to bodily and psychosocial changes following such interventions.

Studies in the field of critical gender studies have shed some light on how gender constructions and embodiment interplay in the case of bariatric surgery. In an interview-based study conducted from a feminist perspective, Newhook et al. (2015) revealed striking differences between how men and women narrated their embodied experiences *before* undergoing bariatric surgery. Whereas women talked about their sense of identity as fat women and their ongoing efforts to compensate for their sense of shame at having a fat body, men focused on their identity as big guys, emphasizing their lack of discomfort with bodily “bigness,” provided it was associated with masculine muscularity. However, Newhook and colleagues did not use aspects of masculinity as an analytical lens; rather their focus was on the gendered meanings embedded in women's narratives, as contrasted with those of men. Although both male and female respondents were interviewed prior to surgery, the study did not elaborate on the men's post-surgery narratives.

Phenomenologically oriented studies on bariatric surgery have also focused primarily on women's bodily experiences, highlighting how excess skin, intestinal problems, and chronic pain problems post-surgery can give rise to discrepancies between women's private and social lives (Groven, Råheim, & Engelsrud, 2010; Groven, Råheim, & Engelsrud, 2013; Natvik, Gjengedal, Moltu, & Råheim, 2014; Murray, 2009). However, one recent study focused on men's bodily experiences of bariatric surgery. In their phenomenologically inspired study, Natvik, Gjengedal, Moltu, and Råheim (2015) explored men's health and well-being 5 years after they had undergone surgery. Their principal finding was that dramatic weight loss enabled the men to become proactive agents in their new lives, regaining opportunities to live unrestricted, independent lives. However, some of the men were found to have suffered complications and severe side effects, which challenged their efforts to live more healthy lives. Although the analysis was not conducted within a gendered paradigm, the study highlighted some of the potential challenges bariatric surgery can present to men's sense of self and masculine identity. In this study, we seek to shed further light on the intersection between bariatric surgery and gender by exploring men's bodily changes following bariatric surgery. Specifically, we ask:

How do men experience and make sense of their bodily changes following bariatric surgery?

## Theoretical framework

In our study, the central theoretical point of departure is Connell's concept of *hegemonic masculinity*. This concept revolves around hierarchical forms of masculinity and how variations of masculinity form a major analytical lens for research (Connell, 1995). According to this framework, the dominant or culturally authoritative form of masculinity in Western culture—what it means to be a “real” man in any given context—is characterized by ideals such as power, success, strength, stoicism, and self-sufficiency. Those who do not conform to hegemonic masculinity, by for example belonging to “marginalized” or stigmatized groups such as ethnic minorities and homosexuals, are seen to be discriminated against. This in turn works to drive men's desire to “live up to” hegemonic masculine ideals in their everyday lives (Connell, 1995; Farrimond, 2011).

According to Connell, hegemonic masculinity is intimately related to bodies: “true masculinity” is almost always thought to proceed from men's bodies (Connell, 1995). From within a hegemonic masculine perspective, men's physique has tended to be conceptualized in terms of a healthy, well-functioning, and muscular body that is able to complete tasks and fulfill normative roles (e.g., father, worker, mate, etc.): so-called pragmatic embodiment (McCreary, Saucier, & Courtenay, 2005; Watson, 2000). Men who embody hegemonic masculinity express their desire for a well-functioning body for the purposes of work, sport, and everyday life (Jackson & Lyons, 2012).

More recent work on embodied masculinity has uncovered the complexities and contradictions that often exist in the relationship between men and their bodies. Along with functionality, men also place value on body shape and appearance, both of which have been shown to play a significant role in influencing men's experiences of health and illness (Robertson, 2006a, 2006b). Such research is relevant to our focus, since bariatric surgery involves radical changes in both bodily appearance and bodily functioning—changes that are likely to impact on men's relations with others as well as their perceptions of themselves.

In order to fully understand men's experiences of bodily changes following bariatric surgery we have also found it relevant to draw on Leder's phenomenological concepts of *dys-appearance* and *disappearance*. When an individual is healthy and feeling well, the body tends to disappear in the sense that it recedes in the background of awareness. By contrast, in situations of more or less dramatic bodily change, the body tends to dys-appear in the sense of being experienced as something that stands in the way as an obstinate force interfering with the individual's projects (Leder, 1990, p. 84; Groven, 2014; Groven, Råheim, & Engelsrud, 2013). According to Leder, dys-appearance involves both social and bodily dimensions. Situations involving the critical or objectifying gaze of others, for example, are likely to affect an individual's self-consciousness (sense of self) and sense of others (Leder, 1990, p. 96). Through the application of a phenomenological perspective, we acknowledge that men undergoing bariatric surgery are situated in a particular sociocultural context (Merleau-Ponty, 2002; Leder, 1990). This perspective enables us to explore men's experiences of bodily changes, a process we see as inevitably intertwined with, and potentially informed by, (hegemonic) norms of masculinity.

## Methods

From the outset, we regarded interviews as the appropriate method for exploring men's experiences following weight-loss surgery. Individual interviews are particularly useful when the researcher seeks to develop in-depth knowledge concerning an individual's experiences. In addition, interviews are a highly relevant method for developing knowledge in fields where qualitative methods have not been used extensively (Kvale & Brinkman, 2009). In the following section, we elaborate on the interview process, our analytical process, and the ethical issues we addressed. We also provide details of our sample and how we recruited participants.

### Participants

This study was approved by the Norwegian Regional Committee for Medical and Health Research Ethics. It draws on the experiences of five Norwegian men, all of whom have undergone a gastric bypass operation. Information about the study was provided to potential participants, along with a letter asking if they would be willing to join the study. The letter emphasized that their participation was voluntary and that they could withdraw at any time during the study. Once the men had signed the informed consent form we ensured their confidentiality, utilizing fictitious names and omitting identifying details such as age, social status, and family background (Kvale & Brinkman, 2009).

The first author recruited participants through a support group and an organization offering help and guidance to individuals undergoing various kinds of obesity treatment, including bariatric surgery. In addition to having undergone gastric bypass surgery, participants also had to have undergone the surgery more than 4 years previously, given our focus on men's long-term experiences—a dimension largely underrepresented in the literature owing to more women presenting for bariatric procedures.

All participants were aged 24–46 and of ethnic Norwegian origin. However, they represented a varied sample in terms of demographic background, marital status, education, and work experience.

One participant, “Eirik,” had been married for more than 15 years. “Sebastian” and “Kristoffer” had both been divorced, in Sebastian's case prior to his undergoing bariatric surgery and in Kristoffer's case after the procedure (Sebastian had remarried by the time of the interview, but Kristoffer was still single). “Brage,” too, was single, although he admitted to dating once in a while. “Vegar” was in a steady relationship. Two of the men had children. At the time of the interviews, all five men were in full-time employment, working in various areas (farming, food and catering, health services, management, and office work). All but one of the men had changed jobs after undergoing surgery. All five participants had struggled with obesity for several years prior to undergoing bariatric surgery. They had undergone their gastric bypass surgery in different hospitals in Norway. Two of the men had paid for the surgery themselves in private clinics, whereas the remaining three had undergone surgery in public hospitals with all costs covered. Following acute, life-threatening complications Vegar, Sebastian, and Kristoffer had undergone several subsequent operations.

### Interviews

The first author conducted individual interviews with all five men. Taking a semi-structured approach, she encouraged the men to elaborate on various aspects of their life situation prior to and following the surgery. According to her thematic interview guide, she focused on asking open questions to encourage the men to elaborate on experiences they found significant.

During the first part of the interview, she asked the men to describe their life situation prior to bariatric surgery, including their experiences as “obese” men. She also encouraged them to talk about their experiences with food, dieting, physical activity, and exercise. Throughout, the interviewer tried to remain an active listener, encouraging the participants to dwell on their experiences and occasionally posing questions for the purposes of clarification. In addition, participants were asked to give concrete examples of episodes when it seemed relevant (Van Manen, 1997, p. 67). This approach elicited many details concerning various episodes and situations that had informed the men's decisions to undergo gastric bypass surgery. It also offered insights into their reflections on these circumstances.

A similar approach characterized the second part of the interview, which focused on participants’ lives post-surgery.

The men talked at considerable length about the “positive” outcomes of the surgery, including their dramatic weight loss and changed lifestyle. However, when asked to view in retrospect some of the challenges they had encountered since their surgery more than 4 years earlier, the men spoke of severe complications, including repetitive episodes of internal herniation, which had impacted their lives in dramatic and unforeseen ways. In other words, once the men were invited to talk about the more problematic aspects of their post-surgical experience (although the word *problematic* was replaced by *challenge*), they went into considerable detail. This inspired the interviewer to pose various follow-up questions, in order to gain a deeper understanding of these challenges.

In terms of methodology, this experience of probing men's health experiences, particularly those described as having a “positive result,” resonates with the findings of other research suggesting that men's hesitation to talk about health and illness hinders the work of researchers and clinicians alike (Ahlsen, 2014).

### Analysis

Following Braun and Clarke's (2006) recommendation that the point of departure for thematic analyses should be congruent with how one conceptualizes the subject matter, we began by jointly reading through the interviews in order to identify potential themes, in particular those that seemed to involve the interplay of lived experiences with norms of masculinity. One aspect that soon came to our attention was that participants’ desire for a “normal life” was closely intertwined with their experiences of life prior to their surgery, particularly if these involved risk of chronic disease and/or social exclusion. We found it interesting that participants explicitly articulated experiences of being socially excluded. The use of phrases such as “hoping for a better life” and “my life is completely changed compared to previously” seemed to represent a type of temporality often found in narratives of illness (Riessman, 2008) and prompted us to label this theme *getting a “normal life.”* When exploring *how* the men specifically described a normal life we entered a new, more complicated layer of surgery-related experiences, ones that in most cases had taken place more than 2 years post-surgery. We labelled this theme *dealing with ongoing bodily changes*. Finally, we identified a theme revolving around the men's ongoing efforts to balance social and bodily aspects following bariatric surgery: *a better, but more complicated life*.

## Methodological considerations

Our emphasis on in-depth interviews provided nuanced and complex insights into the men's long-term experiences of bodily changes following bariatric surgery. Moreover, interviewing men who had all undergone the same operation (gastric bypass) helped shed light on the challenges associated with this procedure.

The fact that we interviewed participants more than 4 years after their surgery opens the possibility that their experiences were shaped by the passage of time as well as by their present life situation. It could also be argued that, having already undergone surgery, participants were perhaps likely to justify and defend their earlier decision. This tendency became particularly clear to us when the men emphasized their absence of regrets: all of them said they would undergo surgery again, despite their struggles to deal with various side effects and complications.

Because all of the men included in the study were ethnically Norwegian, their homogeneity could be considered a limitation in terms of transferability. However, it should be noted that the participants varied in terms of age, educational background, and social status, and they had undergone surgery in various hospital settings, private as well as public. The cultural setting, especially with regards to how masculinities are constructed and negotiated within a Norwegian context, represents a unique aspect of our study and one that needs to be taken into account when considering the transferability of our findings. We have sought throughout to relate our findings to those of studies conducted in other contexts. Based on this comparison to other contexts, we would argue that although hegemonic masculinity intersects with several potential social structures such as ethnicity, religion, and sexuality, we believe, in line with Connell (1995), that a healthy, well-functioning, and muscular body appears as a key tenet of hegemonic norms of masculinity in Western countries.

After the first author conducted the interviews and transcribed them, all three authors participated in the analysis of the empirical material. This group effort allowed us to identify various themes and discuss them both in light of our phenomenological framework and through a gender-sensitive perspective. These themes were identified as offering fruitful perspectives that, in combination, could shed light on the men's experiences of bodily change. Moreover, given that we were a mixed group of researchers in terms of gender (two female researchers and one male researcher) and nationality (two Norwegian and one British) the analysis process included ongoing discussions of alternative interpretations before an agreement was reached, allowing us to achieve both distance from and closeness to our own preconceptions and thereby to enhance the trustworthiness of our findings.

## Findings

### Getting a “normal” life

Profoundly evident in the men's accounts were the severely restricted social lives they had experienced prior to their surgery. They told of how their large, heavy bodies had prevented them from living the active, fulfilling social lives they desired. For some men, it was the physical restrictions their weight placed on their capabilities that hampered their ability to live a normal life. Others had experienced feelings of shame at not being able to lose weight on a permanent basis, and these feelings had cast a shadow over their lives, leading them to withdraw from social activities outside the home. Eirik, a husband and father, recalled feelings of tiredness, frustration, and having little desire or willingness to play with his children or engage in activities outside the home. He preferred instead to stay on the sofa:

I had sleep apnea and was snoring something awful. My wife was very bothered; she had great difficulty sleeping because of me and had to sleep with earplugs …. And life was hard work. It was most problematic in terms of getting out. So I had no real obesity-related disease, but it was mentally exhausting. And you could not stand so much, and did not bother so much.

Eirik had struggled with being overweight since early childhood and spoke candidly about how he had been bullied, both as a child at school and as an adult in the working environment:

Actually, everyone at my work commented on it … me being fat. I was … my colleagues called me “the fat guy” …. And in a male-dominated workplace, it is kind of a rough style, and in such a climate being called the fat guy is normal …. Instead of saying hello, they called you the fat guy … it is kind of a jargon …. And it was like that all the time.

Being overweight also impacted participants’ relationships with friends. Sebastian, for example, recalled how his friends suddenly stopped inviting him on hunting trips, which he attributed to their doubts about his ability to cope with the physical demands involved on account of his obesity:

They stopped taking me hunting and stuff because they were afraid that I might die or something like that, I think.

Most men had struggled with depression for long periods prior to their surgery. Vegar spoke of battling with emotional eating since early childhood, recalling how he had been “in and out” of the psychiatric hospital over a period of several years. Similarly, Kristoffer had suffered with depression during his adolescent years, in addition to what he labeled “overeating.” By contrast, Brage emphasized that during his adolescent years he had lived an active, healthy life, taking part in wrestling on a regular basis. However, food became increasingly problematic and he reached a point where he would eat “early and late, you eat when you can.”

For all the men in our study, the dramatic weight loss that occurred during the first 2 years following bariatric surgery was perceived as a positive turning point and the beginning of a more “normal” life. Engagement in the activities they felt they had missed out on during their years of being overweight became increasingly possible. Physical activity, in particular, became a major focus in most men's “new” lives as they progressively lost weight. Fewer practical and social barriers were now evident; participants described being able to find appropriate clothing and no longer being worried about what others might think of them at the gym.

Four years after their surgery, most participants were still exercising on a regular basis. Brage's regular exercise included spinning (cycling in the gym) and weight lifting. He described how over time he had become aware of how being slimmer and fitter was influencing his self-esteem and perception of his body and its capabilities:

Of course you appreciate the change. Wow, here is actually a muscle that I have not seen before. And that's what's fun about exercising, too. Then one senses having made progress.

Vegar also talked about how regular exercise had dramatically improved his self-esteem and overall outlook on life:

I'm very satisfied. I put a lot of work into building my body …. Not just for … not just for health, but also to build my self-image …. Create more sense of achievement. And then everything good in life comes from that.

Kristoffer had begun cycling to work on a daily basis. During the interview he talked of how the surgery, combined with his active change of lifestyle, had enabled him to become what he considered to be a normal weight:

My self-esteem has improved as I have lost more and more weight …. And I sense that people relate to me differently now that I appear to be of normal weight. Cycling to work in my cycle gear every day regardless, all year long … I appear to be an exercise person, right.

### Dealing with ongoing bodily changes

Dramatic weight loss enabled participants to overcome previous restrictions, changing their life for the better in many ways. They were able to move more freely around, be active, and take part in exercise on a regular basis, as well as enjoy positive comments from others with regard to their slimmer and more fit appearance. At the same time, their post-surgical lives involved being alert to ongoing bodily changes.

The men's bodies were constantly changing, both on the inside and on the surface. Kristoffer had undergone three operations to remove excess skin and was on the waiting list for three more surgical procedures. However the ugly scars and complications that had resulted from these earlier procedures had made him worried about the upcoming ones. He had also had to take sick leave for several months and avoid exercise during his recovery process:

It's quite a big operation. It gets to me, being sedentary for a period afterwards …. And then I have to re-start exercising, which is hard, certainly no pleasure …. After two of the operations I went up in weight and struggled to get back to life … so it takes a toll on you both physically and mentally. The body is tired of being under anesthesia … and being operated on.

Although the scars did not bother him in his daily life, he experienced them as problematic when dating new partners or wearing shorts on holiday. Eirik, by contrast, was not considering having plastic surgery to remove excess skin. Having regained a significant amount of the weight he had lost, he did not regard his (excess) skin as being so problematic as to require surgery. As he put it, he did not feel the need to look like the professional footballer Ronaldo:

I realize that if I was to get a tight abdomen and stuff like that I had to [have the extra skin removed]. But I'm married and have kids so I have no need to look like Ronaldo. And I do not have any big plans about getting divorced and going back on the market.

In addition to surgical procedures to remove excess skin, three of the men had also undergone acute surgery due to severe complications. Vegar's experience was particularly stark. After undergoing acute surgery to correct internal bleeding, he began to suffer from ulcers in the first week after the operation. During the two subsequent years, he suffered four further acute, life-threatening episodes of internal herniation and had to undergo several more operations:

It really began right away … I just arrived home and was sent back to the hospital because I got bleeding and leakage …. So then I had another operation. Then I got ulcers, but it went OK. So it took … well a year then … before … I got something additional, which was internal herniation. And after a year I got internal herniation twice … then I went a year again and then I got more internal herniation.

Vegar also had to undergo three more surgical interventions as a result of complications following the surgical removal of his excess skin. Four years after undergoing bariatric surgery, he emphasized his struggle with fatigue and concentration, which made it difficult for him to focus at work. Prior to undergoing bariatric surgery Vegar had considered himself to be a “workhorse”; after it, he had found it difficult to endure even a day at work. He described how he had been on sick leave several times during the previous year and expressed fear that this might lead to him losing his job:

And it's very sad because it seriously affects my everyday work …. It's actually a bit comical because yesterday I got a letter about it … saying they [his employer] are very dissatisfied with how I … with my working capacity compared to previously. So I have been summoned to a negotiation meeting. So it is, yes … bloody annoying, sorry for the expression.

Participants who had experienced internal herniation all spoke of it as being very dramatic and emotionally scarring, a situation sometimes compounded by the failure of medical staff to immediately recognize the severity of the men's condition and the men's reluctance to express the intensity of their pain. Sebastian described it thus:

The first doctor I told you about … he just laughed at me. “You are supposed to have pain,” he said. Well … I can tolerate a lot of pain and manage it. Perhaps I did not manage to express how painful it really was, I recommend that everyone who experiences anything like that should roll around and play dead on the floor or … at least not leave out any symptoms …. Because there's too little knowledge about this matter.

Like Sebastian, Kristoffer also experienced episodes of internal herniation. When recalling his first episode, he emphasized the creeping, aversive, and intensifying nature of the pain:

It's a creeping kind of pain, because it starts to hurt, and then it develops into pulsating pain, sort of like a roof that is blowing off. And then there come peaks of pain, which only get worse and worse and worse. And then the peaks last longer and longer, more and more painful. And finally you are lying down there, screaming …. Then it's so painful that when the peaks come you're incapable of dealing with the pain at all.

As this extract highlights, Kristoffer's pain was so intense that he felt that it was beyond his control. Unable to move, even to go to the toilet, he knew that something was seriously wrong inside his body:

I realized it when I woke up in the morning …. This is completely wrong, this is not ordinary stomach pain, this is not obstipation or something like that.

Through his involvement with an Internet support group for individuals undergoing bariatric surgery Kristoffer was reminded of how fortunate he had been in his situation, for others had had to have parts of their intestine removed due to complications such as internal herniation. However, Kristoffer continued to experience problems following his two episodes of internal herniation and was strongly of the opinion that the repeated episodes of constipation he had suffered were a direct consequence of his several surgical interventions:

But that is for sure! Those surgeries did something about the way my stomach works, as it often turns into constipation.

Sebastian's main post-operative challenge was dealing with repeated episodes of hypoglycemia, which had started 2 years after his bariatric surgery and were totally unexpected:

The first time I experienced it I recall having eaten a lot and was on the field with my tractor. And I do not recall driving home again or anything, so that … I was nearly gone.

On a subsequent occasion he woke up in the middle of the night with diarrhea and his tongue nearly bitten off. While he had been informed about internal herniation prior to his surgery, he was not prepared for hypoglycemia. It came completely unexpectedly and for a while he wondered, given the symptoms he was having, whether he was suffering from epilepsy or diabetes:

Actually, when you are having hypoglycemia it feels like you have been drinking a bottle of aquavit …. You become foggy and then you become destructive and irritated, and you … when you go far enough into hypoglycemia then … then you don't understand what you are doing, frankly speaking. You are not able to see clearly, you become numb. It is exactly the same as an epileptic seizure or hyperglycemia, which is … diabetes … exactly the same.

Sebastian gradually found ways to self-manage the frequency and intensity of these hypoglycemic episodes. One method was to always carry licorice with him:

One always has to carry licorice in a pocket, along with a sandwich. And when I sense it coming I grab a piece of licorice. It is like having diabetes. When you have diabetes, you don't go anywhere without sugar. I take licorice before I start to feel muzzy. I can feel it in my body when it's about to come. I become so numb and shaky and, yes, so I usually know whether I have done something wrong then, if I have eaten too much sandwich, eaten two sandwiches instead of one.

Through experience, Sebastian had also learned that there were finely drawn limits to how much licorice he could actually eat. Indeed, the consequences of not following a strict diet could be fatal. On one occasion, Sebastian collapsed while driving, leading to his driving license being revoked^3^:

And occasionally I eat too much licorice [laughs] …. It might happen that I eat licorice when I am not supposed to eat it … I had to go through a bunch of tests the following year and finally, I got it back. But it was not the last time … it was during my first fainting that I lost my driver's license. For then I got really scared. I thought I had epilepsy and went to see my doctor …. So my advice is not to tell anyone, and just do things right.

Sebastian's desire to independently manage the hypoglycemia episodes associated with “late dumping” was clearly evident in his account. Although others may have considered it to be dramatic seeing him laying down shivering during a hypoglycemic episode, he had learned from previous experience that his symptoms would pass as long as he relaxed and managed to eat some licorice or sugar. For Sebastian, maintaining his driving license was key to his independence and he had made the decision not to discuss his “late dumping” episodes with his doctor due to the fear that his license might be revoked again. Experience had taught him that he had to “just do things right,” and while he recognized that the amount of licorice that he ate may not be approved by his doctor, he was confident that he had found a way to manage it himself.

### A better, but complicated, life

Although side effects and complications represented ongoing challenges in the men's new lives, most regarded their surgery as successful. Losing dramatic amounts of weight had changed their life situations in many positive ways, enabling them to live more socially active and fulfilling lives. This situation contrasted with the restricted lives they had led prior to surgery. As Vegar put it:

I'd say that I'm a good success story. I have experienced a huge change, a unique change.

Reflecting on their experiences, the men emphasized that their achievements were made possible through their own efforts. They saw their ongoing lifestyle changes, rather than the surgery, as the key to their success. As Kristoffer put it:

I'm very focused on getting the message across that I have not lost weight because of the surgery, I have lost weight because of the lasting lifestyle changes I have managed to make. It's this massive change that I've made in terms of lifestyle that has enabled me to keep my weight down and stay changed. So, it's not the surgery in itself.

In a similar vein, Vegar identified his (new) exercise habits, now intertwined with his daily routines, as the most significant reason for his health and well-being:

I'm on the same level as a professional athlete, at least that's what my doctor says. I feel that is exaggerating it a bit …. Though I am very active, I exercise very hard and often. I am very strong and my heart function is very good … I run … yes … half-marathon … my entire lifestyle is completely changed. And that's what it is all about. Actually changing your lifestyle, making the road you are walking healthy and sustainable for the rest of your life. I don't think so much about it anymore, I just do what I have to do, because now it has become a matter of habit.

Sebastian described the surgery as a form of “antabus.” Six years after his gastric bypass surgery he enjoyed being “fitter than ever”; he had stabilized his body weight and was now enjoying going hunting, chopping wood, and working on his farm:

The surgery in itself is just an antabus. You have to change your lifestyle … I've never been fitter than I am now. I'm not into marathons and stuff like that, but it works very well for me now. Both in terms of work, hunting, and everything, that is … I cannot complain.

At the same time, Sebastian experienced his energy level as somewhat reduced, preventing him from exercising as intensely and frequently as he would have liked. He reasoned this had less to do with the surgery than with his eagerness to engage in too many projects:

I wish I could manage to exercise and stuff like that, but I don't have the energy for that … I exercise by chopping wood and hunting, and I don't have any surplus for anything more than that.

Eirik had a contrasting experience to relate: that of regaining some of his old weight. Now 4 years after the surgery, this weight gain was having obvious consequences for his bodily appearance: the enlarged breasts and beer belly he had developed prior to surgery now were becoming visible again:

Now the flabby chest and beer belly are coming back … me being fat again … psychologically, it's a downturn that this is happening now …. So I just have to pull myself together and sharpen myself up a bit, so that I can get [the weight] off again.

While Eirik found regaining weight psychologically challenging, he also described feeling more relaxed now than previously because he felt more in control of the situation. Being in control meant that he had to “pull himself together” in terms of staying focused on regular exercise and a strict diet:

It is still a daily struggle, I mean that life is still the way it was when I was fat. … No matter, although you've had the surgery … you have to follow the food rules and eat very little food … If you … manage through 1 week to limit your food intake considerably, then the body works well again and then you get rid of the surplus kilos … you can do a lot about it in 2 weeks.

## Discussion

### Surgery as a means of escaping social dys-appearance

Our findings reveal how participants’ sense of self changed radically following surgery. In particular, the men's sense of self-esteem improved as they became slimmer and fitter. This interrelatedness of body and self-esteem can be seen in relation to their sense of being marginalized prior to their surgery, a time when they were subject to critical comments and looks from others. What made the men particularly sensitive to such critical attention was the fact that they were significantly larger and heavier than most other men. This experience resonates with Leder's concept of social dys-appearance, something likely to occur when the gaze of the other is experienced as particularly antagonistic or objectifying, triggering disruption in both social interaction and corporal self-consciousness.

Repetitive experiences of being bullied, excluded, and stigmatized in various social settings and situations demonstrated the men's vulnerability to other men's critical gaze when it came to matters of corporality and fitness. In the same way that chronically ill or disabled men have been found to be “socially emasculated” because they lack the resources required to sustain a hegemonic masculinity, the obese bodies of the men in our study were seen by others as displaying a vulnerability or “weakness of the physical body-self.” Like a disability, this was judged to be incompatible with a “real” (hegemonic) man: one capable of performing and completing tasks (Robertson, Sheikh, & Moore, 2010, p. 701; Shakespeare, 1999, p. 54)

Of particular significance in the men's accounts following bariatric surgery was the extent to which their bodies were intertwined with their masculine identity. However, while some of their experiences appeared informed by hegemonic masculinity, others did not reflect this masculine ideal. For example, the men emphasized that once they had made up their mind to take action to reduce their weight, they were proactive about seeking surgery. For some of the men, this involved paying for surgery out of their own pockets: they opted for treatment in private clinics so as to get it done as soon as possible and without having to wait in line for a publicly funded surgery. Although they did not suffer from obesity-related diseases, such as diabetes or cardiovascular disease, they were well aware that they could get ill if they did not lose weight on a permanent basis. Opting for treatment in private clinics can thus be interpreted as a modern response to the risks associated with illness (Bayer, 2008; Chrysanthou, 2002; Gard & Wright, 2001; Galdas, 2009) as well as a late-modern masculine way of taking responsibility for one's shape and appearance (Robertson, 2006a, 2006b).

### A new masculine lifestyle

Engaging in a more active and masculine lifestyle was a central part of the men's new lives. Participants spoke at length about their new exercise habits, which included cycling, fitness training, and running marathons. For Kristoffer, achieving a normal weight and cycling to work changed his status from fat person to “exercise guy.” As for the men undergoing rehabilitation following heart attacks in a study by Robertson et al. (2010), this active lifestyle facilitated participants’ engagement in daily activities.

Our findings also highlight how the men's efforts to live more active lives were intimately intertwined with their fear of regaining weight. They emphasized that engaging in regular exercise was what was enabling them to control their body weight more than 4 years after their gastric bypass surgery—a period when the risk of regaining weight is particularly high (Hofsø, 2011; Natvik 2015). A closer inspection of Eirik's experiences revealed that his awareness of the reappearance of enlarged breasts and a beer belly when he put weight back on had resulted in him “pulling himself together” and exercising more frequently and intensely. This connection suggests that engaging in regular exercise was also significant to the men's bodily appearance and not simply to their body's functionality. This line of argument tallies with research indicating that male bodies are increasingly being represented as objects to be viewed, with men increasingly seen as responsible for the appearance and maintenance of their bodies (Liechty, Ribeiro, Sveinson, & Dahlstrom, 2014). Pressure to achieve a bodily ideal has also been related to a discourse linking weakness and lack of control to being overweight, which by this token may constitute a threat to masculinity (Grogan & Richards, 2002). However, it should be noted that Eirik differed from the other men we interviewed because of his openly expressed fear of regaining weight and his daily struggle to prevent this from happening. Other participants emphasized their success in terms of weight loss, which they saw as enabling an active lifestyle.

Although hegemonic masculinity in Norway has traditionally placed an emphasis on outdoor life and sport-related activities, displaying sportiness and fitness while competing with other men has become growingly popular over recent years (Dahl-Michelsen, 2015; Hervik & Fasting, 2014). This is particularly evident in sports competitions such as the Birkebeineren, a cross-country ski event that attracts men from all over the country (more than 80% of participants in the Birkebeineren are male).^4^ After the race, the media are particularly keen on publishing the results of prominent Norwegian men, including celebrities and managers in some of Norway's leading financial concerns.^5^ ^6^

The status associated with participating and doing well in such competitions, and the fact that the vast majority of those participating there are men, underline the priority now being given to keeping fit, being athletic, and having the grit to endure pain and discomfort for extended periods. Such elements appear central to the construction of embodied hegemonic masculinity in Norway. If this is the case, one could argue that Vegar's sporty, competitive lifestyle embodies a more hegemonic masculinity than the one presented in Eirik's account. From being marginalized as an obese man and prevented from living the life he desired, Vegar had moved up the hierarchy: along with a fitter body, he now enjoyed social recognition as a sporty, competitive man. Vegar's way of displaying masculinity was, in other words, sensed and lived by endurance sports, in which he could now compete with other men. These findings resonate with Drummond's notion that enduring physical pain is a central part of what it means to be a man. More precisely, he argues that successful involvement in endurance races such as triathlons is perceived as setting men apart from one another, thereby creating a *hierarchy* of masculinities (Drummond, 2010). This is not to argue that men not engaged in competitive sports are not displaying hegemonic masculinity. In their study of Norwegian men's notions of their own bodies, Hervik and Fastings (2014) found that outdoor activities involving the functionality of men's bodies also connoted hegemonic masculinity norms. In our study, some reported experiences intersect with this line of thought: for example, engaging in hunting and chopping wood, as opposed to running marathons.

In sum, by losing dramatic amounts of weight as well as engaging in various types of exercise and physical activity, participants had in many respects moved up the social hierarchy: they were displaying enhanced hegemonic masculinity when compared with the past. At the same time, fear of regaining weight was placing this enhanced hegemonic masculinity under constant threat.

### The struggle to maintain balance

Although the men tried their best to display a hegemonic masculine lifestyle and sense of self, they could not ignore embodied side effects and complications. Due to acute and chronic complications they found themselves negotiating an ongoing tension between feeling ill and being healthy. Learning to live with this tension involved acknowledging their bodily experiences as a source of insight—insight that both threatened and facilitated their masculine identity. Acknowledging their bodily experiences as a source of insight often meant learning the hard way: through testing, failing, and living on the edge, as part of a risky, inevitable balancing process.

For the participants, intense pain in their viscera could prove life-threatening if they failed to take prompt action. They learned that the pain associated with internal herniation, rather than passing off, would spread throughout the body: a pulsating, intolerable pain so intense and aversive that it was impossible to suppress or control. As noted by Leder (1990), in such a situation the body *dys-appears*, in the sense of manifesting itself in the foreground of one's attention. The person is ceaselessly reminded of the here-and-now body. Pain that cannot be removed, Leder suggests, exerts a *telic demand* upon the sufferer, who seeks to find a means of being free of pain:

My own body becomes the object not just of perception and interpretation but of action. I seek medication, physical therapies whatever will help. My projects are reorganized around and attempt to cope with or remove the pain. Instead of just acting from the body I act toward it. (Leder, 1990, pp. 78–79)

Although the men in our study quickly reasoned that they had to get professional help to get rid of their pain, until they got surgical help the pain intensified. In Leder's terms, this placed an “affective call” upon them (Leder, 1990, p. 73). In such a situation, not being taken seriously by health professionals was experienced as frustrating. On the basis of his experience, Sebastian advised others suffering from internal herniation after bariatric surgery to exaggerate the pain rather than put on a brave face, since the latter would risk not being taken seriously by the doctor examining them. At the same time, Sebastian emphasized that he could usually “tolerate a lot of pain and manage it.” This sentiment ties in with traditional hegemonic masculinity norms on the significance of being stoical and enduring pain no matter how intense (Ahlsen, 2014). Through experience, Sebastian learned that pain associated with internal herniation could and should not be endured. He thereby emphasized the importance of acknowledging his bodily experiences and doing what was necessary to get help, rather than trying to be brave and remain poised on the edge. We see this aspect as also illuminating how bodily experiences intersect with masculine norms regarding making sense of pain and illness. It also points to the possibility of participants challenging traditional norms of masculinity as they strive to recover from severe, potentially life-threatening complications (Ahlsen, Mengshoel, & Solbrække, 2012).

Traditional masculinity norms—in particular norms related to bodily discipline, independence, and enduring pain—were also negotiated during episodes of hypoglycemia. During one of his first episodes of hypoglycemia, which occurred nearly 2 years after his bariatric surgery, Sebastian fainted and everything went black. Reflecting on this episode, he emphasized how devastating it had been for him to lose his driver's license. During the following months he found himself dependent on others, a new situation for a man given to fixing everything without help. This account suggests that masculine norms regarding remaining independent and in control were at stake. At the same time, Sebastian spoke in detail about how he had learned to prevent further seizures from happening. Thanks to these efforts, he had learned that knowing when to draw the line was crucial, since the consequences of crossing the line could be fatal. Occasionally, he deliberately crossed that line by eating more licorice than he should. The way he joked and laughed about this in the interview suggests that, for him, living on the edge could in some ways be exciting. Or was it, perhaps, that for him humor was the only socially acceptable way of dealing with the challenge, as a recent study on men's ways of dealing with cancer suggests (Roaldsen, Sørlie, & Lorem, 2015)?

Such behavior can be interpreted as a means of challenging limits by occasionally living on the edge. Again, this idea accords with traditional masculinity norms. The notion of edgework, introduced by Collinson in 1996, illuminates male offenders’ involvement in risky activities as a means of performing dominant masculinity. According to Robertson (2007), this emphasis on risk-taking can be interpreted as a need to transcend the banality of everyday life. In the case of our research, edgework emerges as a way of coping with the restrictions imposed on the men's viscera and food intake by gastric bypass surgery. For participants, taking risks once in a while can be seen to represent a means of demonstrating independence and control and thus hegemonic masculinity norms. This tallies with Robertson's view that modern men tend to negotiate between “should care” and “don't care” as a means of maintaining their hegemonic male identity (Natvik, Gjengedal, Moltu, & Råheim 2015; Robertson, 2006b). For the men in our study, “risky” eating was particularly associated with social settings involving friends, girlfriends, wives, and family. This suggests that their efforts to make sense of side effects and complications in their quest for living healthy and socially rewarding lives also involved a desire to eat like “ordinary” men.

Previous studies on men and dieting have found that “real men” do not diet: dieting is first and foremost regarded as a feminine undertaking (Gough, 2007; Gough & Conner, 2006). Research also indicates that men tend to see exercise as a more masculine model of weight control than food restriction and diet modification (Drummond, 2010, p. 204). For the participants in our study, dietary changes were partly enforced on them as a result of surgery. Due to the restricted size of their stomach and their decreased food absorption, they could no longer eat as they had done in the past. They had to make radical changes to the type of food they ate and the quantities in which this was consumed.

### Implications for practice

By analyzing men's accounts of their experience following gastric bypass surgery, this study highlights the importance of placing men's recovery efforts within a broad framework of understanding. In particular, our findings point toward the need to understand men's individual efforts to make sense of their life after bariatric surgery, including their changed and changing body, as a process of trying, failing, and testing various limits. It should also be recognized that such efforts are intertwined with hegemonic masculinity ideals. Those providing advice and support for men in similar situations may wish to encourage their patients to be attentive to the signals from their body after surgery. They may also be advised to openly discuss any experiences that they consider to be problematic or that appear to contradict medical advice.

Finally, health professionals may wish to pay closer attention to the role played by exercise and physical activity in helping male patients adjust to a changed situation, including that following bariatric surgery. As our study reveals, eating continued to pose challenges for our participants and their masculine identities, even years after surgery. For men in a similar situation, exercise and physical activity may well be a means of strengthening their self-esteem, including their masculine sense of self. Physical activity may also help men cope better with daily life, even in situations where their capacities have been dramatically reduced in the wake of complications and side effects. We recommend that the potential of physical activity and exercise for men's rehabilitation following bariatric surgery be explored further in future studies.

Finally, since the majority of those undergoing bariatric surgery are women, our study has revealed an area that needs further research: the exploration of men's own experiences (understood as intimately intertwined with norms of masculinity) so that health professionals may be able to more fully understand the needs of males who undergo bariatric surgery.

Further research understanding the gendered nature of recovery and rehabilitation post-bariatric surgery through samples of men therefore appears important.

## Footnotes

1
www.who.int/mediacentre/factsheets/fs311/en/

2 Surgical treatment for obesity. www.kvalitetogprioriteringer.no/saker/kirurgisk-behandling-ved-overvekt

3 Hypolgycemia—also termed *late dumping*—differs from early dumping in the sense that it is associated with an exaggerated release of insulin, causing symptoms similar to those of diabetics (Kulick et al., 2010).

4
www.birkebeiner.no/no/Nyhetsartikler/Hvorfor-konkurrerer-kvinner-mindre-enn-menn/

5 Disse kjendisene går birken (these celebrities are competing in the Birkebeiner race). www.e24.no/livsstil/birkebeinerrennet-2015/disse-kjendisene-gaar-birken/23416524 Advarer mot birkebeinerlederskal (warning against Birkebeiner leadership). www.stage.ukeavisenledelse.no/nyheter/ledelse/advarer-mot-birken-lederskap

6 Sosiolog: Det er status blant ledere å være veltrent (Sociologist: Status among leaders to exercise and be fit). www.e24.no/jobb/sosiolog-det-er-status-blant-ledere-aa-vaere-veltrent/3475201
